# TIGAR promotes neural stem cell differentiation through acetyl-CoA-mediated histone acetylation

**DOI:** 10.1038/s41419-019-1434-3

**Published:** 2019-02-27

**Authors:** Wenjuan Zhou, Tiantian Zhao, Jingyi Du, Guangyu Ji, Xinyue Li, Shufang Ji, Wenyu Tian, Xu Wang, Aijun Hao

**Affiliations:** Key Laboratory of the Ministry of Education for Experimental Teratology, Shandong Provincial Key Laboratory of Mental Disorders, Department of Human Anatomy and Histoembryology, School of Basic Medical Sciences, Shandong University, Jinan, Shandong China

## Abstract

Cellular metabolism plays a crucial role in controlling the proliferation, differentiation, and quiescence of neural stem cells (NSCs). The metabolic transition from aerobic glycolysis to oxidative phosphorylation has been regarded as a hallmark of neuronal differentiation. Understanding what triggers metabolism reprogramming and how glucose metabolism directs NSC differentiation may provide new insight into the regenerative potential of the brain. TP53 inducible glycolysis and apoptosis regulator (TIGAR) is an endogenous inhibitor of glycolysis and is highly expressed in mature neurons. However, its function in embryonic NSCs has not yet been explored. In this study, we aimed to investigate the precise roles of TIGAR in NSCs and the possible involvement of metabolic reprogramming in the TIGAR regulatory network. We observed that TIGAR is significantly increased during brain development as neural differentiation proceeds, especially at the peak of NSC differentiation (E14.5–E16.5). In cultured NSCs, knockdown of TIGAR reduced the expression of microtubule-associated protein 2 (MAP2), neuron-specific class III beta-tubulin (Tuj1), glial fibrillary acidic protein (GFAP), Ngn1, and NeuroD1, and enhanced the expression of REST, suggesting that TIGAR is an important regulator of NSC differentiation. Furthermore, TIGAR enhanced the expression of lactate dehydrogenase B (LDHB) and the mitochondrial biogenesis and oxidative phosphorylation (OXPHOS) markers, peroxisome proliferator-activated receptor gamma coactivator 1 (PGC-1α), nuclear respiratory factor (NRF1), and MitoNEET during NSC differentiation. TIGAR can decrease lactate production and accelerate oxygen consumption and ATP generation to maintain a high rate of OXPHOS in differentiated NSCs. Interestingly, knockdown of TIGAR decreased the level of acetyl-CoA and H3K9 acetylation at the promoters of *Ngn1*, *Neurod1*, and *Gfap*. Acetate, a precursor of acetyl-CoA, increased the level of H3K9 acetylation and rescued the effect of TIGAR deficiency on NSC differentiation. Together, our data demonstrated that TIGAR promotes metabolic reprogramming and regulates NSC differentiation through an epigenetic mechanism.

## Introduction

NSCs are defined by their capacity for self-renewal and for differentiation into neurons, astrocytes, and oligodendrocytes^1^. NSCs can generate new neurons in the course of normal physiological activity in both developing and adult mammalian brains to guarantee the integrity and precision of neural connection and cerebral function. However, in most neurodegenerative diseases and most brain trauma, neuronal damage is irreversible; the neuron population cannot be refreshed through NSC differentiation. A full understanding of the differentiation potential of NSCs may help us apply cell-replacement therapeutic strategies against neurological diseases^2,3^. Growing evidence has shown that multiple metabolic pathways participate in cell fate determination in NSCs^4^. Metabolic dysfunction is closely associated with many neurological diseases, including schizophrenia as well as Alzheimer’s (AD), Parkinson’s (PD), and Huntington’s diseases (HD)^5^. Given the important role of metabolism in regulating the proliferation and differentiation of NSCs, pharmacological intervention in metabolic homeostasis may be able to exploit the regenerative potential of NSCs against these neurodegenerative diseases.

TIGAR is generally regarded as an antiapoptotic gene expressed in response to p53-induced cell death. As a bisphosphatase, TIGAR reduces intracellular fructose-2,6-bisphosphate levels, resulting in an inhibition of glycolysis^6^. TIGAR is highly expressed in human breast carcinoma cells. Recent evidence showed that overexpression of TIGAR in carcinoma cells altered metabolic compartmentalization to a mitochondrial metabolic phenotype and ultimately increased tumor growth^7^. Importantly, TIGAR is widely distributed in neurons and plays crucial roles in the central nervous system (CNS). For example, in ischemic stroke, TIGAR protects against ischemic/reperfusion-induced injury via glucose 6-phosphate dehydrogenase-enhanced pentose phosphate pathway (PPP) flux^8^. However, in the developing brain, the effect of TIGAR in NSCs is largely unknown.

Glucose is the obligatory energy substrate in the brain. The regulation of glucose metabolism in the CNS is a key process that has been investigated for many years^9,10^. Studies have confirmed that NSCs rely mainly on aerobic glycolysis to maintain their energy supply, while mature neurons depend on oxidative phosphorylation^4^. Reduced levels of oxygen enhance the survival and proliferation of cultured NSCs and inhibit differentiation. NSCs also display higher lactate dehydrogenase activity, more lactate production, and higher glucose consumption than cultured neurons^11^. Importantly, metabolic reprogramming from aerobic glycolysis to oxidative phosphorylation (OXPHOS) has been found during neuronal differentiation. Decreased expression of hexokinase (HK2) and lactate dehydrogenase A (LDHA), and a switch in pyruvate kinase gene splicing from PKM2 to PKM1 were found during NSC differentiation^12^. However, it remains unclear what conditions can trigger metabolic transition and how metabolism regulates neuronal differentiation. It is well known that OXPHOS can supply more energy than glycolysis during neuronal differentiation to enable protein synthesis and neuronal activity. The role of metabolites produced during glucose metabolism cannot be ignored during cell fate decisions of NSCs.

As mentioned, TIGAR is an important endogenous inhibitor of glycolysis. Whether TIGAR participates in metabolic reprogramming during NSC differentiation is unknown. In this study, we explored the effect of TIGAR in NSCs and investigated the potential molecular mechanism of TIGAR-induced NSC differentiation. Fundamental understanding of the metabolic mechanisms of TIGAR in NSCs may help provide new therapies that reactivate neurogenesis to treat degenerative diseases.

## Material and methods

### Primary NSC culture

Primary NSCs were isolated and cultured as previously described^13,14^. Briefly, brains were obtained from Kun Ming mice at embryonic day 12.5 (E12.5). Then, the brains were cut mechanically and digested with 0.25% trypsin (Gibco, USA) for 5 min at 37 °C. Dulbecco’s modified Eagle’s medium (DMEM)/F12 (1:1) medium (Gibco, USA) was used to neutralize the effect of trypsin. After centrifugation, NSCs were resuspended and 2×10^5^/ml cells were seeded in 25-cm^2^ T-flasks with DMEM/F12 medium supplemented with 2% B27 (Gibco, USA), 20 ng/ml bFGF (R&D, USA), 20 ng/ml epidermal grown factor (EGF; Invitrogen, USA), and 1% penicillin/streptomycin solution. Cells were incubated at 37 °C under a 5% CO_2_ atmosphere for five days. Then, the primary neurospheres were collected and digested using trypsin and EDTA, and incubated in serum-free medium for another 3–5 days (passage 1 neurospheres). To investigate NSC differentiation, we dissociated passage 1 neurospheres, stimulated them with differentiation medium containing 2% fetal bovine serum (Gibco, USA) without growth factors, and cultured them for 1–9 days. All animal experiments complied with the National Institutes of Health Guide for the Care and Use of Laboratory Animals and were approved by the Institutional Animal Care and Use Committees of Shandong University (no. 201402020).

### RNA interference plasmid constructs and lentivirus packaging

Plasmid constructs were designed as previously described^15^. The *Tigar* shRNA forward sequences 5′-TTA GCA GCC AGC ATC TTA GTT CAA GAG ACT AAG ATG CTG GCT GCT AAT TTT TT-3′ and reverse sequences 5′-AAT TAA AAA ATT AGC AGC CAG CAT CTT AGT CTC TTG AAC TAA GAT GCT GGC TGC TAA GGC C-3′ were annealed and ligated into pSilencer 1.0 vector. Then the U6 promoter and the *Tigar* shRNA sequences were cut from pSilencer 1.0 and inserted into PGW vector. The PGW plasmid was a lentiviral transfer vector and contained a *Gfp* reporter gene. The pUltra plasmid was used to package *Tigar* or *Tigar-TM* mutant lentivirus. In addition, three package plasmids pMDL/pRRE, VSV-G, and pRSV-REV were used in the experiments. To produce high titer lentiviruses, these above plasmids were transfected into 293 T cells.

### RNA isolation and real-time quantitative PCR

TRIZOL reagent (Invitrogen, Carlsbad, CA, USA) was used to isolate total RNA from cultured NSCs. The purity and concentration of total RNA was measured by a spectrophotometer. Then cDNA were synthesized with a RevertAid^TM^ First Strand cDNA Synthesis Kit (Thermo Fisher Scientific). Real-time PCR was performed with SYBR Green Realtime PCR Master Mix (TOYOBO CO., Ltd., Japan). The expression of β-actin was regarded as a normalization control, and the 2^−ΔΔCT^ method was used to calculate changes of the gene expression levels. The primer sequences were listed in Table 1.

**Table 1:** primer sequence

Gene	Forward primer	Reverse primer
*Tigar*	AGGGCAGAGAGAAAGCGT	TGCCACCTTTGGGATTC
*Nestin*	CCACAGTGCCCAGTTCTA	ATAGAGTGGTGAGGGTTGAG
*Sox2*	ACTCCATGACCAGCTCGCAGAC	CCTCGGACTTGACCACAG
*Gfap*	AACAACCTGGCTGCGTAT	ACTGCCTCGTATTGAGTGC
*Map2*	GAATAAGCAAGAGCCCAGAG	GTCCGTCGTGCTGAAGAG
*Neurod1*	CTCAGCATCAATGGCAACTTC	CACCGGAAGAGAAGATTGATC
*Rest*	CATGGCCTTAACCAACGACAT	CGACCAGGTAATCGCAGCAG
*Ngn1*	AGTAGTCCCTCGGCTTCAGA	ATGAAACAGGGCGTCGTG
*Ki67*	AAGAGTGAGGGAATGCCTAT	TCATTTGTCCTCGGTGGC
*Glut1*	CTGGGAATCGTCGTTGG	GCAGAAGGGCAACAGGATA
*Glut3*	GCTGGGCATCGTTGTTG	CTGTAGCTTGGTCTTCCTCCT
*Mct1*	GAGGTCCTATCAGCAGTATCTT	CCAGTGGTCGCTTCTTGT
*Mct2*	CACTGGCTCCTTTCAATC	CTGGCTTTCTTCAGAGTTG
*Mct4*	ACTTCAACAAGCGTCGCCCTAT	TCAGTCCCTCCGCCTACCTG
*Pfkfb3*	AGCCTCTTGACCCTGATA	TTCTTGCCTCTGCTGGAC
*Ldha*	GTTGGGGTTGGTGCTGTT	ATCTCGCCCTTGAGTTTG
*Ldhb*	TGGACAAGTGGGTATGGC	TTTTCGGAGTCTGGAGGA
*Pgc-1a*	TGGAGCAATAAAGCGAAGA	GCGGTTGTGTATGGGACT
*Nrf1*	TCCCAGAGATGCTCAAGTA	CTATGGTCCGTAATGCCT
*Mitoneet*	AGAGAATCGCACCAAAGC	GTCGCCAGTCTCTTCGTT
*Tomm20*	AGCACAAGGTGACTACGAG	TGACTAATGGTCGGAAGC
*Acly*	ACGTGTGCATCTATGCTAC	GGACCAACAGGTGTCTCTTA
*Acss2*	CCTCTACTGCTTTGTTACCT	GAGCGTGTTTTAGGCAAG
*β-actin*	CGTTGACATCCGTAAAGACCTC	CCACCGATCCACACAGAGTAC
*Ngn1 (ChIP)*	CATTGTTGCGCGCCGTA	GCGATCAGATCAGCTCCT
*Neurod1 (ChIP)*	GTCCGCGGAGTCTCTAACTG	GAACCACGTGACCTGCCTAT
*Ngn1 (ChIP)*	ACAAAAGGCCTGGGTTGACA	CTCTGGATCTGGAACTCGCC

### Immunofluorescence

Cultured NSCs or embryonic brain sections were fixed with 4% paraformaldehyde for 30 min and rinsed with PBS (pH 7.4) three times. Cells or brain sections were blocked with 10% goat serum containing 0.3% Triton X-100 for 2 h and then incubated with primary antibodies overnight at 4 °C. The following primary antibodies were used: rabbit anti-TIGAR (1:500, ab37910; Abcam), mouse anti-Nestin (1:200, ab6142; Abcam), mouse anti-Sox2 (1:200, ab171380; Abcam), mouse anti-Tuj1 (1:200, #4466; CST), mouse anti-GFAP (1:200, BA0056; Boster), and rabbit anti-GFP (1:200, #2956; CST). After being washed three times in PBS, the cells or slices were incubated with secondary antibodies conjugated to FITC or TRITC for 1 h at room temperature. The sections were stained with 2 µg/ml 4′,6-diamidino-2- phenylindole (DAPI) before being photographed. Images were obtained with an IX71 Olympus fluorescence microscope. 5-Ethynyl-2′-deoxyuridine (EdU) labeling assays were detected using a Cell-Light EdU Apollo 488 In Vitro kit (Ribobio, Guangzhou, China). NSCs cultured in differentiation medium for 5 days were treated with 50 μM EdU for 2 h according to the manufacturer’s protocol. Then, cultured NSCs were fixed with 4% paraformaldehyde for EdU staining. The number of EdU- or DAPI-positive cells was counted with ImageJ. TUNEL staining was performed using the Fluorescein In Situ Apoptosis Detection Kit (KeyGEN BioTECH, China).

### Western blotting

In vitro cultured NSCs or mouse embryonic cortical tissue was homogenized in RIPA buffer (Beyotime Institute of Biotechnology, Shanghai, China) containing protease and phosphatase inhibitors for 30 min at 4 °C. After centrifugation at 12000 rpm at 4 °C for 15 min, the supernatants were collected. The protein concentration was measured using the Pierce bicinchoninic acid (BCA) assay kit. The samples were mixed with 5× loading buffer (Boster, Wuhan, China), and equal amounts of protein were loaded into each well of 8–15% SDS-PAGE gels. Then, the separated proteins were transferred to PVDF membranes (Bio-Rad). After being blocked with 5% milk in TBST buffer for 2 h, the membranes were incubated with primary antibodies at 4 °C overnight and then with HRP conjugated secondary antibodies for 1 h at RT. After being washed with TBST buffer, blots were visualized using an enhanced chemiluminescence (ECL) kit (Merck Millipore). Primary antibodies were used at the following dilutions: rabbit anti-TIGAR (1:1000, ab37910; Abcam), rabbit anti-β-actin (1:2000, #4970; CST), mouse anti-Tuj1 (1:1000, #4466; CST), mouse anti-GFAP (1:1000, BA0056; Boster), mouse anti-GLUT1 (1:500, sc-377228; Santa), mouse anti-MCT1 (1:500, sc-365501; Santa), mouse anti-LDHA (1:500, sc-137243; Santa), mouse anti-LDHB (1:500, sc-100775; Santa), rabbit anti-H3K9ac (1:1000, #9649; CST), rabbit anti-H3K18ac (1:1000, #9675; CST), rabbit anti-H3K14ac (1:1000, #7627; CST), rabbit anti-H3K27ac (1:1000, #4353; CST), and rabbit anti-H3 (1:1000, #4499; CST).

### Mitochondrial isolation and acetyl-CoA quantification

A mitochondria isolation kit (ab110170, Abcam) was used in this experiment^16,17^. In brief, cultured NSCs were frozen and thawed to weaken the membranes and suspended in Reagent A. Then, the cells were homogenized and centrifuged at 1000× *g* at 4 °C for 10 min. The supernatant was collected (SN1), and the pellet was resuspended with Reagent B. After homogenization and centrifugation, the supernatant was collected again (SN2). SN1 and SN2 were mixed thoroughly and centrifuged at 12,000× *g* for 15 min at 4 °C. The pellet was collected in Reagent C supplemented with protease inhibitors and used for acetyl-CoA quantification.

Acetyl-CoA quantification was conducted using an acetyl-CoA assay kit (Solarbio life sciences, Beijing, China). Briefly, 5×10^6^ cells or isolated mitochondria were collected and incubated in extraction buffer for 30 min. The cells were subjected to sonication and centrifuged at 8000× *g* at 4 °C for 10 min. The supernatants were collected and supplemented with acetyl-CoA assay buffer. The 340 nm absorbance values were measured at 20 s (A_20s_) and 80 s (A_80s_). The difference between A_80S_ and A_20S_ was used to calculate the relative level of acetyl-CoA.

### Fluorescent glucose uptake assay, lactate production assay, and ATP measurement

Cultured cells were rinsed with Krebs-Ringer-HEPES (KRH) buffer and incubated for 30 min in the presence of 600 μM 2-[N-(7-nitrobenz-2-oxa-1,3-diazol-4-yl)amino] −2-deoxyglucose (2-NBDG), a fluorescent glucose tracer used to measure glucose transport^18,19^. Then, the cells were washed with PBS to remove the unabsorbed 2-NBDG and fixed with 4% paraformaldehyde. The accumulation of 2-NBDG in cells was imaged by a fluorescence microscopy. NIH ImageJ software was used to calculate the fluorescence value of each group. Ten non-overlapping images were analyzed in each group.

Lactate release was measured using a lactate assay kit (1200011002, Eton Bioscience), and ATP was measured using a luminescent ATP detection assay kit (ab113849, Abcam) according to the manufacturer’s protocol.

### Oxygen consumption rate assay

For analysis of mitochondrial respiratory potential, Seahorse Bioscience XFe 96 Extracellular Flux Analyzer was used to monitor Oxygen consumption rate (OCR) in differentiated NSCs according to the manufacturer’s instructions. 8×10^4^ cells per well were seeded on PDL-coated XFe 96 plates, and incubated at 37 °C in 5% CO_2_. The NSCs were transfected with Lenti-*Gfp* or Lenti-*Tigar* for 72 h. On the day of detection, the cells were washed with XF basal medium containing 17.5 mM glucose and 1 mM sodium pyruvate (pH 7.4) and incubated at 37 °C in a non-CO_2_ incubator for 1 h. OCR was measured with sequential injections of 1 μM oligomycin, 0.5 μM FCCP and 0.5 μM each of rotenone/antimycin A. OCR was normalized to the protein concentration in each well.

### Chromatin immunoprecipitation assay

Chromatin immunoprecipitation (ChIP) was conducted using an EZ-ChIP kit (Merck Millipore). Cultured NSCs were treated with 1% formaldehyde for 15 min at RT to generate cross-links between histones and DNA. Then, the cells were washed three times with PBS and collected in SDS lysis buffer supplemented with protease inhibitors. After sonication, the chromatin in each cell lysate sample was sheared into 200–500 bp fragments. The supernatants were immunoprecipitated with a specific antibody (anti-H3K9ac, CST) or a control antibody (anti-IgG) overnight at 4 °C. Then, the supernatants were subjected to washing, elution and cross-link reversal following the manufacturer’s instructions. Purified genomic DNA from the supernatants was analyzed using real-time PCR. The primers were listed in Table 1.

### Statistical analysis

Statistical analysis was performed using SPSS program (version 19.0). Data statistical significance was calculated by Student’s *t*-test or one-way or two-way repeated ANOVA, followed by the LSD or Dunnett’s T3 post hoc test. Values were presented as the mean ± SEM and significance was set at *p* < 0.05.

## Results

### TIGAR is highly expressed in the embryonic neocortex and NSCs

**Fig. 1:**
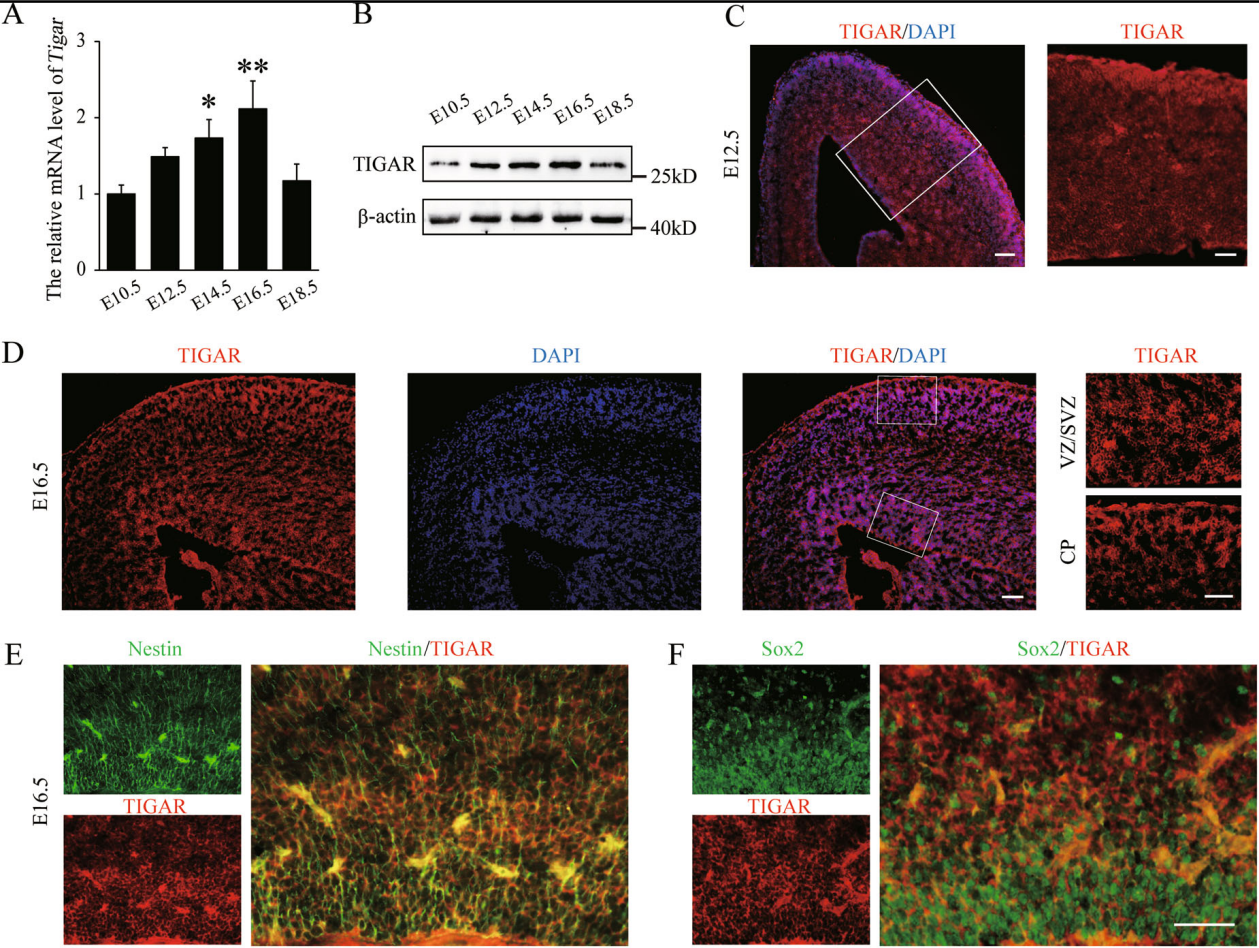
TIGAR expression in the developing neocortex. **a**, **b** QPCR and western blot analysis of TIGAR in the mouse cerebral cortex during embryonic development. β-Actin was used as a control (*n* = 6–8 per group; error bars represent the SEM; *p *<* 0.05, **p *<* 0.01 versus the E10.5 group; one-way ANOVA). **c** Immunofluorescent staining of TIGAR expression in the E12.5 cerebral cortex. Coronal sections were labeled with TIGAR and DAPI. High-magnification image of TIGAR in the cerebral cortex on the right side. Left: scale bar = 100 μm; right: scale bar = 50 μm. **d** Immunostaining of TIGAR expression in the E16.5 cerebral cortex. Scale bar = 100 μm. Right panel: high-magnification image shows high expression of TIGAR in the VZ/SVZ and CP. VZ/SVZ ventricular zone/subventricular zone, CP cortical plate. Scale bar = 50 μm. **e**, **f** Double immunofluorescent staining shows that TIGAR is expressed in the NSCs in the VZ/SVZ at E16.5. **e** Double immunofluorescent staining of TIGAR (red) and Nestin (green). **f** Double immunofluorescent staining of TIGAR (red) and Sox2 (green). Nestin and Sox2 are two markers of neural stem cells. Scale bar = 50 μm

To investigate the role of TIGAR in brain development, we assessed the expression pattern of TIGAR from E10.5 to E18.5 during embryonic cortex development. Both qRT-PCR and western blot results showed that the expression of TIGAR was significantly increased during cortical development, especially during the peak of NSC differentiation (E14.5 and E16.5) (Fig. 1a, b). Similar results were obtained in immunostaining experiments, and TIGAR was widely distributed in the embryonic cortex, as indicated by its localization in the ventricular zone (VZ), the subventricular zone (SVZ) and the cortical plate (CP) at E16.5 (Fig. 1c, d). *Nestin* and *Sox2* are two markers of NSCs. By immunofluorescent staining for TIGAR and Nestin or Sox2, we found that TIGAR is localized in the cytoplasm of NSCs (Fig. 1e, f). Studies have demonstrated that the time from E12.5 to E16.5 is an important period for the initiation of neuronal differentiation^20^. The data showing that the expression levels of TIGAR increased over the course of neural differentiation suggested that TIGAR may play crucial roles in neural differentiation during embryonic cortex development.

**Fig. 2:**
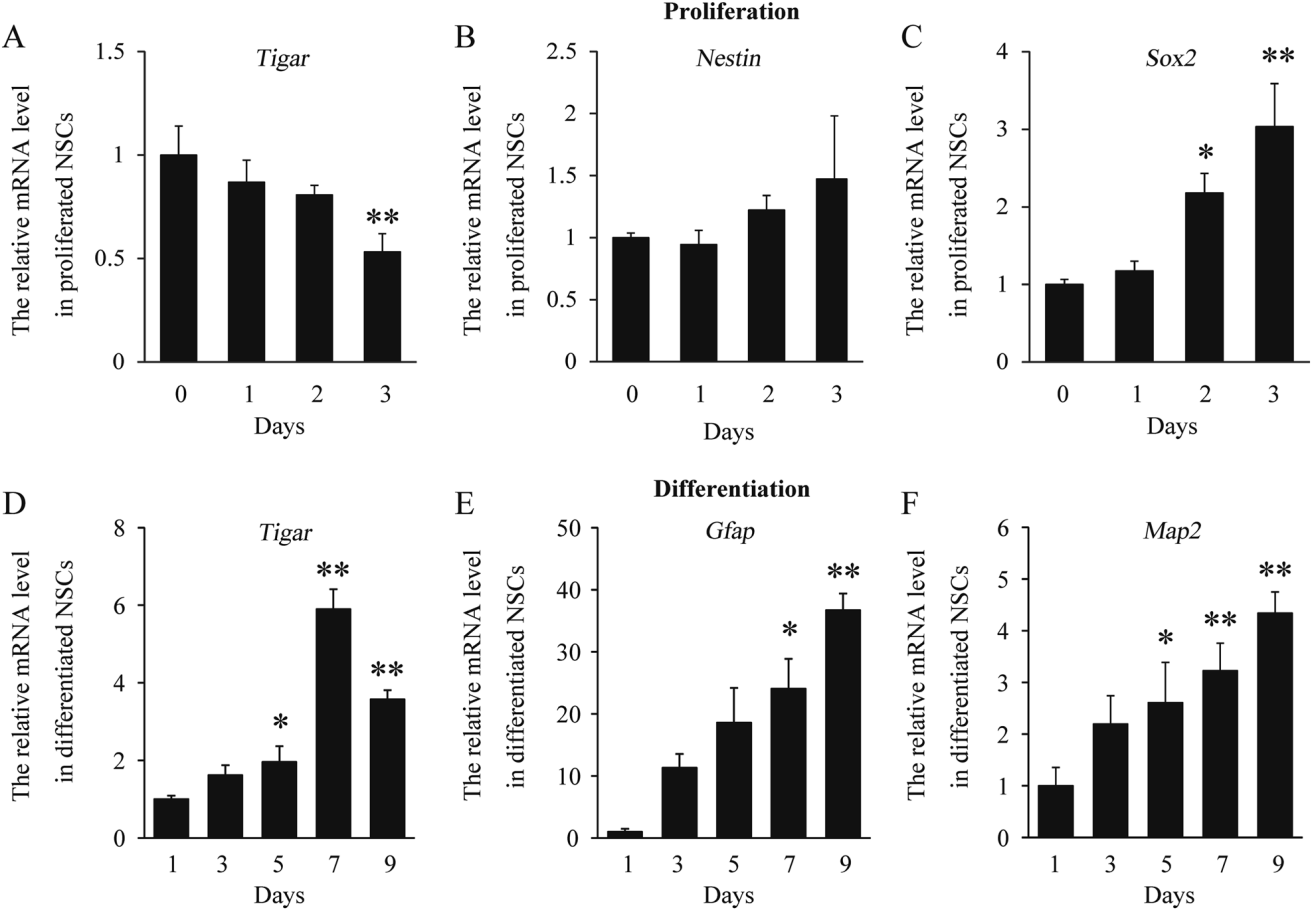
TIGAR is specifically increased during differentiation of NSCs. **a**–**c** Quantification of the mRNA levels of *Tigar*, *Nestin*, and *Sox2* during proliferation of NSCs. **d**–**f** Quantification of the mRNA levels of *Tigar*, *Gfap* and *Map2* during differentiation of NSCs. *p < 0.05, **p < 0.01, one-way ANOVA. Data represent the mean of at least three independent experiments ± SEM

To evaluate whether TIGAR is involved in the differentiation of NSCs, we further analyzed the expression of TIGAR during the proliferation and differentiation of cultured NSCs. NSCs were isolated and incubated with different media. In line with the expression of *Map2* (a neuronal marker) and *Gfap* (an astrocyte marker), *Tigar* was especially increased during the differentiation of NSCs and, conversely, decreased during proliferation (Fig. 2). These data suggest that TIGAR may be an important regulator of NSC differentiation during embryonic development.

### TIGAR is necessary and sufficient for NSC differentiation

**Fig. 3:**
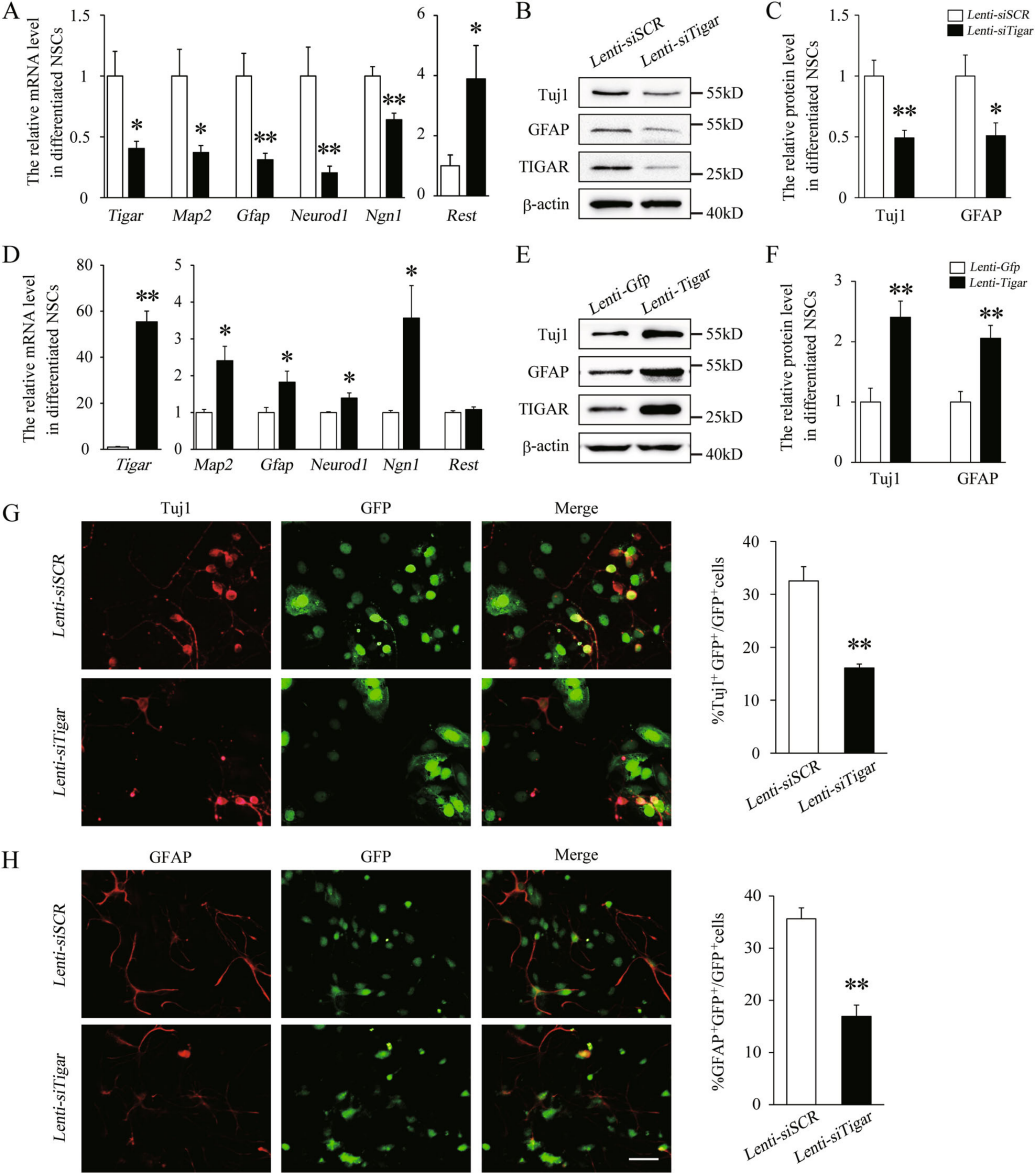
TIGAR regulates the differentiation of NSCs. **a** Quantification of the mRNA levels of *Tigar*, *Map2*, *Gfap*, *Neurod1*, *Ngn1*, and *Rest* in the Lenti-*siSCR*- and Lenti-*siTigar*-treated groups of cultured NSCs. **b**, **c** Representative immunoblots and relative quantification of Tuj1, GFAP, and TIGAR after knockdown of TIGAR in NSCs. **d** Quantification of the mRNA levels of *Tigar*, *Map2*, *Gfap*, *Neurod1*, *Ngn1*, and *Rest* in the Lenti-*Gfp*- and Lenti-*Tigar*-treated groups in cultured NSCs. **e**, **f** Representative immunoblots and relative quantification of Tuj1, GFAP, and TIGAR after overexpression of TIGAR in NSCs. **g** Double immunofluorescent staining showed that knockdown of TIGAR (GFP, green) decreased the expression of Tuj1 (red) and impaired neuronal differentiation of NSCs. The right panel shows the quantitative ratio of Tuj1^+^GFP^+^ to GFP^+^ cells in the Lenti-*siSCR* and Lenti-*siTigar* groups. **h** Double immunofluorescent staining revealed that knockdown of TIGAR (GFP, green) also decreased expression of GFAP (red) and inhibited astrocytes differentiation of NSCs. The right panel shows the quantitative ratio of GFAP^+^GFP^+^ to GFP^+^ cells in the Lenti-*siSCR* and Lenti-*siTigar* groups. Scale bar = 50 μm. *p < 0.05, **p < 0.01, *t*-test. Data represent the mean of at least three independent experiments ± SEM

To further analyze the effect of TIGAR in NSCs, we used modified lentiviruses containing specific sequences to knock down or overexpress *Tigar*. As shown in Fig. 3a, knockdown of TIGAR significantly decreased the mRNA levels of *Map2* and *Gfap* in the differentiation process. It has been reported that the transcriptional activator factors *Ngn1* and *Neurod1* and the repressor factor *Rest* are required for the differentiation of NSCs^21,22^. We found that loss of TIGAR significantly decreased the expression levels of *Ngn1* and *Neurod1* and increased the expression of *Rest* (Fig. 3a). Western blots also revealed a decrease in Tuj1 (a neuronal marker) and GFAP (Fig. 3b, c). These results suggest that TIGAR is necessary for NSC differentiation. In addition, overexpression of TIGAR increased the expression of MAP2, GFAP, Tuj1, NeuroD1, and Ngn1, which suggests that TIGAR is sufficient for NSC differentiation (Fig. 3d–f). In our immunofluorescent staining experiments, GFP-positive cells represent lentivirus-infected NSCs. We found that after stimulation with Lenti-*siTigar* the ratio of Tuj1^+^GFP^+^ to GFP^+^ cells was significantly decreased (Fig. 3g). Lenti-*siTigar* also reduced the ratio of GFAP^+^GFP^+^ to GFP^+^ cells compared with the ratio in the Lenti-*siSCR* treated group (Fig. 3h). Our data revealed that TIGAR can regulate the differentiation of NSCs.

**Fig. 4:**
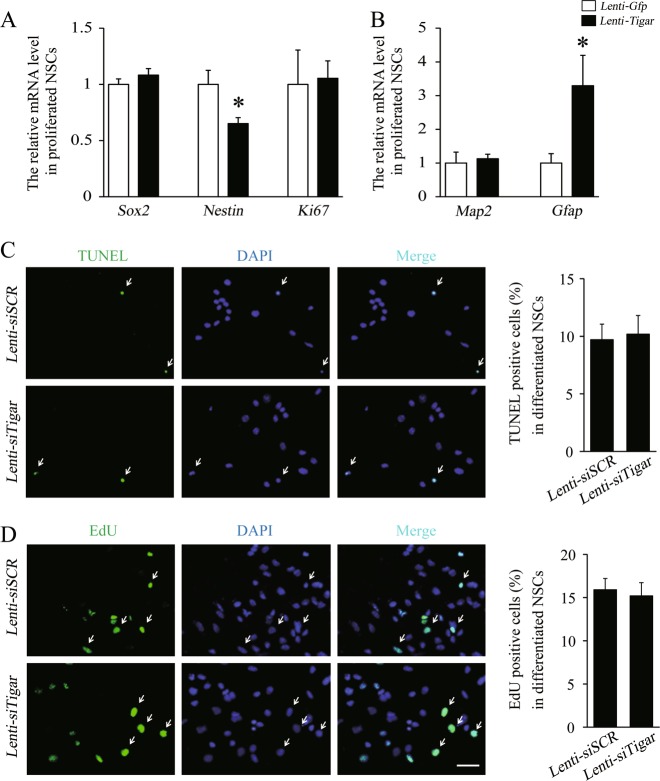
TIGAR has no effect on the cell survival or proliferation of NSCs. **a**, **b** Quantification of the mRNA levels of *Sox2*, *Nestin*, *Ki67*, *Map2*, and *Gfap* in the Lenti-*Gfp*- and Lenti-*Tigar*-treated groups of proliferated NSCs. **c** TUNEL staining (green) in cultured NSCs treated with Lenti-*siSCR* or Lenti-*siTigar* during the differentiation stage. The right panel shows the quantification of TUNEL-positive cells in the Lenti-*siSCR* and Lenti-*siTigar* groups. Scale bar = 50 μm. **d** During the differentiation stage, there was no significant difference in the number of dividing NSCs in the Lenti-*siSCR* and Lenti-*siTigar* groups. The right panel shows the quantification of EdU-positive cells in the Lenti-*siSCR* and Lenti-*siTigar* groups. Scale bar = 50 μm. *p < 0.05, **p < 0.01, *t*-test. Data represent the mean of at least three independent experiments ± SEM

Under proliferation conditions, overexpression of TIGAR decreased the expression of *Nestin* and increased the expression of *Gfap*, but *Map2* was unchanged, suggesting that the role of TIGAR in proliferation is slight and that TIGAR is more likely to accelerate NSC differentiation during differentiated conditions (Fig. 4a, b). We further examined the effect of TIGAR on cell survival and cell proliferation by TUNEL staining and an EdU incorporation assay, respectively, during the differentiation stage. TUNEL staining showed that knockdown of TIGAR had little effect on cell survival (Fig. 4c). The EdU incorporation assay showed that the percentage of EdU-positive cells was similar between the Lenti-*siTigar* and Lenti-*siSCR* treated groups (Fig. 4d). Collectively, our data demonstrate that TIGAR is necessary and sufficient for NSC differentiation.

### TIGAR promoted a shift in glucose metabolism to a mitochondrial phenotype and increased the levels of acetyl-CoA during NSC differentiation

**Fig. 5:**
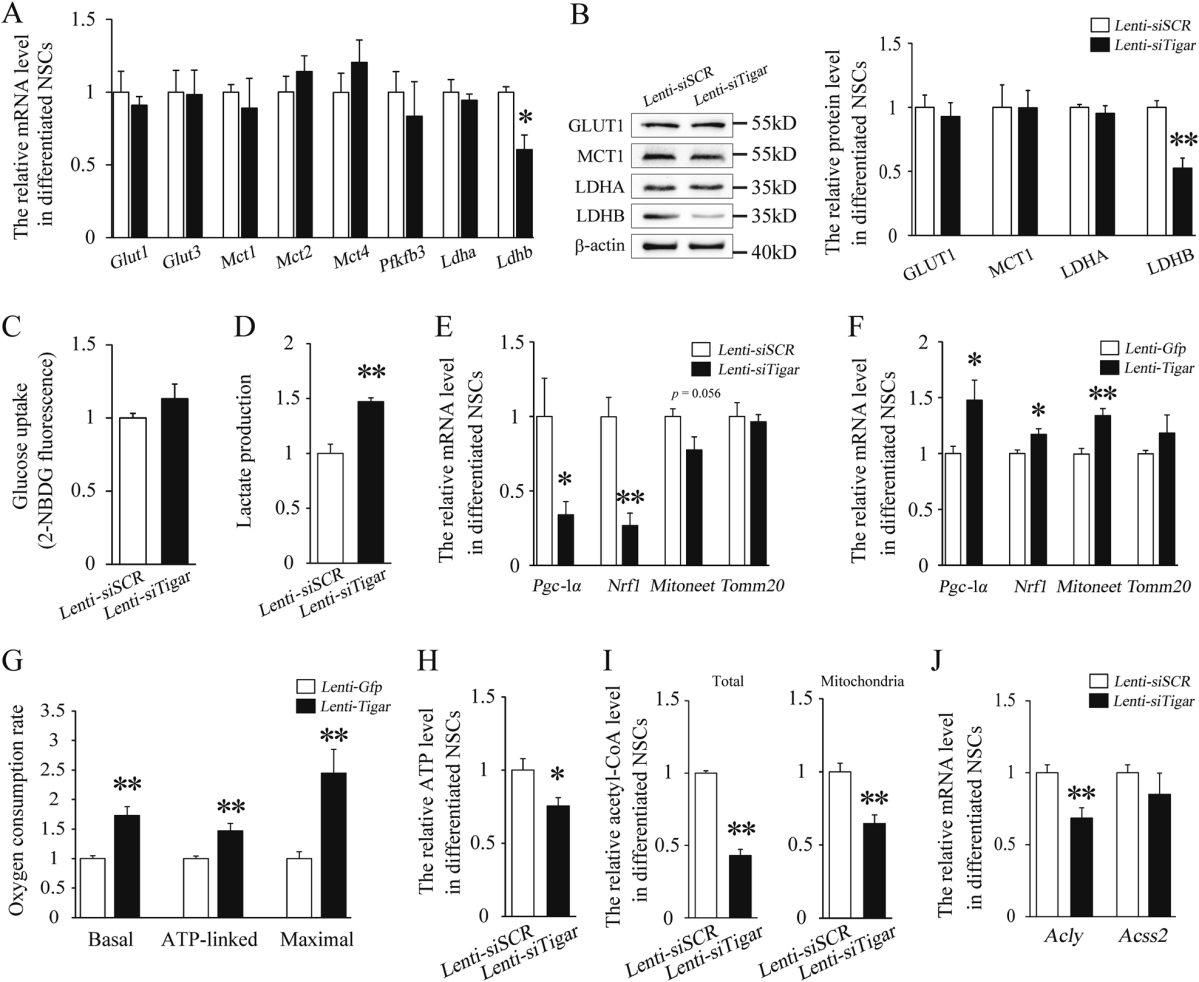
TIGAR induces metabolic reprogramming and increases the level of acetyl-CoA during NSC differentiation. **a** Quantification of the mRNA levels of glycolysis-related transporters and enzymes in the Lenti-*siSCR*- and Lenti-*siTigar*-treated groups in cultured NSCs. **b** Representative immunoblots and relative quantification of GLUT1, MCT1, LDHA, and LDHB in Lenti-*siSCR*- and Lenti-*siTigar*-treated NSCs. **c** Changes in glucose uptake after treatment with Lenti-*siTigar* in cultured NSCs. **d** Lactate production in Lenti-*siSCR*- and Lenti-*siTigar*-treated NSCs. **e** Effect of TIGAR on markers of mitochondrial biogenesis and oxidative phosphorylation. Quantification of the mRNA levels of *Pgc-1α*, *Nrf1*, *Mitoneet*, and *Tomm20* after treatment with Lenti-*siTigar* in cultured NSCs. **f** Quantification of the mRNA levels of *Pgc-1α*, *Nrf1*, *Mitoneet* and *Tomm20* after treatment with Lenti-*Tigar* in cultured NSCs. **g** Oxygen consumption rate (OCR) in Lenti-*Gfp*- and Lenti-*Tigar*-treated NSCs using a Seahorse XFe 96 Extracellular Flux Analyzer. **h** ATP levels in Lenti-*siSCR*- and Lenti-*siTigar*-treated NSCs. **i** Acetyl-CoA production in Lenti-*siSCR*- and Lenti-*siTigar*-treated NSCs. Left: total cell lysates; Right: mitochondrial part. **j** Acetate-dependent acetyl-CoA synthetase 2 (ACSS2) and citrate-dependent ATP-citrate lyase (ACLY) are two principal enzymes that generate acetyl-CoA for histone acetylation. The mRNA level of *Acly* was decreased after knockdown of TIGAR. *p *<* 0.05, **p *<* 0.01, *t*-test. Data represent the mean of at least three independent experiments ± SEM

As mentioned above, TIGAR is an endogenous inhibitor of glycolysis. To investigate the effect of TIGAR on glycolysis in NSCs, we analyzed the expression levels of transporters and metabolic enzymes after knockdown of TIGAR. The results showed that the mRNA and protein expression levels of GLUTs, MCTs, PFKFB3, and LDHA were not changed after stimulation with Lenti-*siTigar* (Fig. 5a, b). However, knockdown of TIGAR decreased the expression of LDHB, which is an important subunit of lactate dehydrogenase that converts lactate into pyruvate for mitochondrial metabolism (Fig. 5a, b). 2-NBDG was used to the measure glucose uptake of NSCs. As shown in Fig. 5c, knockdown of TIGAR had no significant effect on glucose uptake. As expected, the lactate production, as a measure of glycolysis, was increased after knockdown of TIGAR (Fig. 5d). In addition, we examined the expression of the mitochondrial biogenesis markers PGC-1α and NRF1, and the OXPHOS markers MitoNEET and transporter of the outer mitochondrial membrane member 20 (Tomm20) in differentiated NSCs. We found that knockdown of TIGAR decreased the expression of *Pgc-1α*, *Nrf1*, and *Mitoneet*, while overexpression of TIGAR increased the expression of these genes (Fig. 5e, f). To further verify the effect of TIGAR on OXPHOS, oxygen consumption and ATP generation were detected in NSCs. Compared with Lenti-*Gfp-*treated group, overexpression of TIGAR increased the oxygen consumption rate in differentiated NSCs (Fig. 5g). Besides, as shown in Fig. 5h, knockdown of TIGAR significantly decreased the level of ATP. These results suggest that TIGAR may enhance the transition from lactate to pyruvate and promote OXPHOS during NSC differentiation.

Evidence has demonstrated that metabolic reprogramming can induce alteration of cellular acetyl-CoA levels^23,24^. Interestingly, we found that the level of acetyl-CoA in total cell lysate was significantly decreased after TIGAR deficiency (Fig. 5i). After isolation of mitochondria, our data further revealed that knockdown of TIGAR reduced the production of acetyl-CoA in mitochondria (Fig. 5i). After knockdown of TIGAR, the decrease in LDHB expression and mitochondrial activity may cause less pyruvate to become acetyl-CoA in mitochondria. Acetate-dependent acetyl-CoA synthetase 2 (ACSS2) and citrate-dependent ATP-citrate lyase (ACLY) are two important enzymes that participate in the production of acetyl-CoA for histone acetylation^24^. As shown in Fig. 5j, knockdown of TIGAR significantly decreased the mRNA level of *Acly*. It is possible that the decrease in mitochondrial activity and mitochondrial acetyl-CoA level may influence the expression of ACLY. Using protocols from a previous study, we generated the *Tigar-TM* mutant (triple mutant H11A/E102A/H198A) to abolish TIGAR metabolic enzyme activity in fructose bisphosphatase (FBPase-2)^6^. Overexpression of TIGAR-TM mutant lost its effect on glycolysis and OXPHOS (Fig. S1A, B). Importantly, we found that blocking the metabolic enzyme activity of TIGAR abolished its effect on the expression of *Acly* (Fig. S1C). This suggests that the effect of TIGAR on ACLY expression may be related to the regulation of mitochondrial activity and the level of acetyl-CoA in mitochondria. Overall, we concluded that TIGAR enhanced mitochondrial metabolism, and increased the level of acetyl-CoA during NSC differentiation.

### TIGAR regulates NSC differentiation by increasing the level of acetyl-CoA

**Fig. 6:**
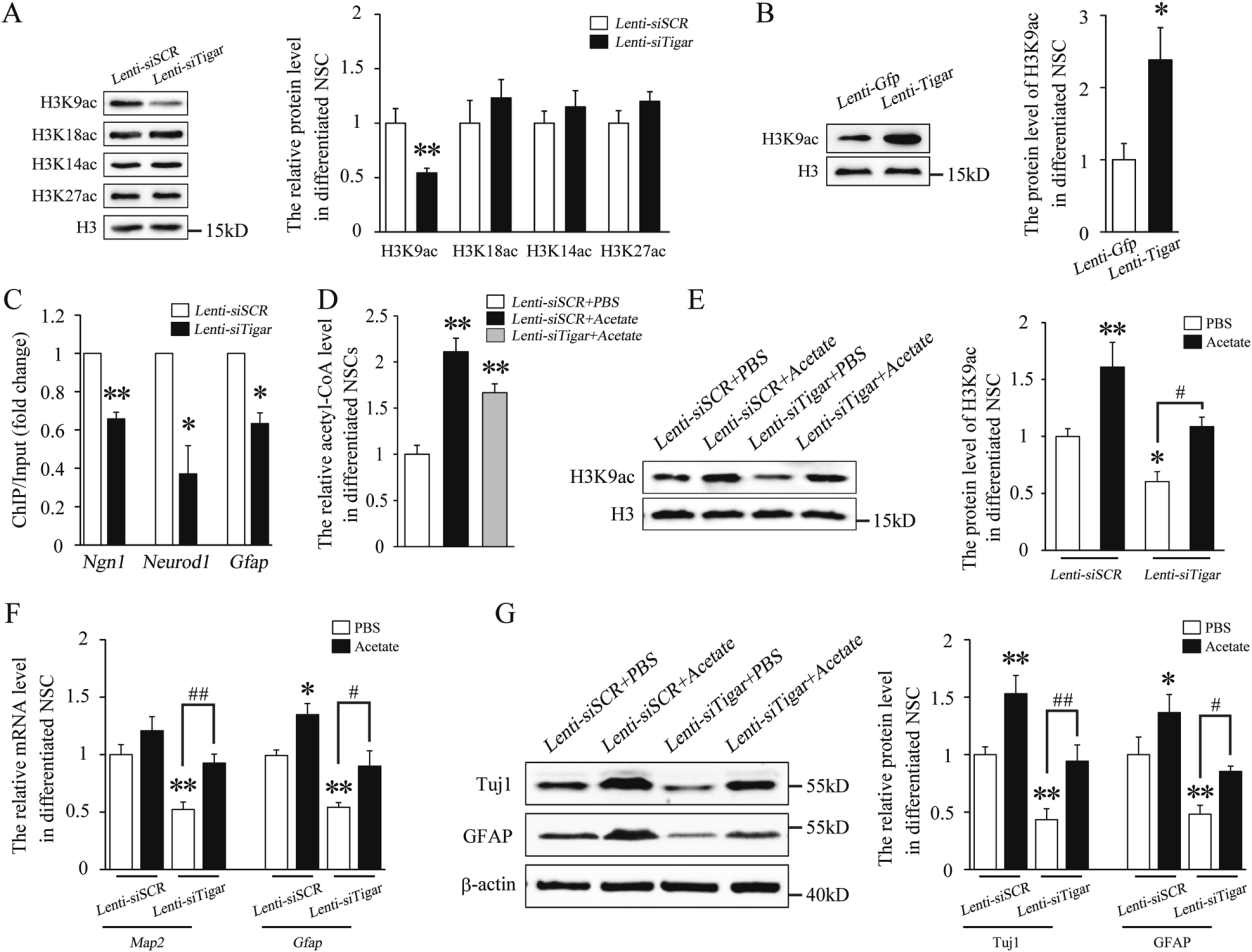
TIGAR promotes NSC differentiation by upregulating the level of acetyl-CoA and the acetylation of H3K9. **a** Western blot analysis of whole-cell lysates showed that H3K9 acetylation was reduced after treatment with Lenti-*siTigar*. **b** Western blot analysis showed that Lenti-*Tigar* significantly increased H3K9 acetylation during NSC differentiation. **c** Lenti-*siTigar* decreased the accumulation of H3K9ac on the promoters of *Ngn1*, *Neurod1*, and *Gfap*. NSCs were immunoprecipitated with anti-acetyl-H3K9 antibody and analyzed using gene-specific ChIP primers. Rabbit IgG was used as a negative control. DNA from each ChIP sample was normalized to the corresponding input sample. **d** Acetyl-CoA production in Lenti-*siSCR*, Lenti-*siSCR* + Acetate, and Lenti-*siTigar* + Acetate group. **e** Western blot analysis showed that supplementation of acetate in cultured medium increased H3K9 acetylation and rescued the effect of Lenti-*siTigar* on the decrease in H3K9 acetylation in NSCs. **f** Quantification of the mRNA levels of *Map2* and *Gfap* after treatment with acetate and Lenti-*siTigar* in cultured NSCs. Acetyl-CoA can be generated from acetate by acetyl-CoA synthetase independently of citrate. **g** Western blot analysis showed that acetate rescued the effect of Lenti-*siTigar* on the decrease in Tuj1 and GFAP protein levels. *p < 0.05, **p < 0.01. Data represent the mean of at least three independent experiments ± SEM

Acetyl-CoA, a central metabolic intermediate, has been shown to be an important second messenger that regulates histone acetylation^23^. Therefore, we tested for changes in histone acetylation in Lenti-*siTigar*-treated NSCs. Our data showed that knockdown of TIGAR did not alter the level of H3K18, H3K14 or H3K27 acetylation (Fig. 6a). However, acetylation of H3K9 was significantly decreased after loss of TIGAR (Fig. 6a). As expected, overexpression of TIGAR increased H3K9 acetylation (Fig. 6b).

Evidence has shown that histone acetylation is an important component of epigenetic regulation to activate gene transcription^25^. Therefore, we asked whether H3K9 acetylation can regulate the expression of NSC differentiation-related genes. Our ChIP results showed that the levels of H3K9 acetylation at the promoters of the *Ngn1*, *Neurod1*, and *Gfap* genes were significantly decreased after knockdown of TIGAR in differentiated NSCs (Fig. 6c). Acetyl-CoA can be generated from acetate by ACSS2, which is independent of citrate^26^. As shown in Fig. 6d, supplementation with acetate reversed Lenti-*siTigar*-induced decrease of acetyl-CoA level. Importantly, acetate significantly increased the level of H3K9 acetylation and rescued the effect of Lenti-*siTigar* on the decrease in H3K9 acetylation in NSCs (Fig. 6e). Furthermore, qPCR and western blotting showed that acetate reversed the decreases in MAP2, Tuj1, and GFAP expression caused by knockdown of TIGAR (Fig. 6f, g). Together, our results suggest that TIGAR may regulate NSC differentiation via increases in acetyl-CoA levels and H3K9 acetylation at the promoters of *Gfap*, *Neurod1*, and *Ngn1*.

## Discussion

**Fig. 7:**
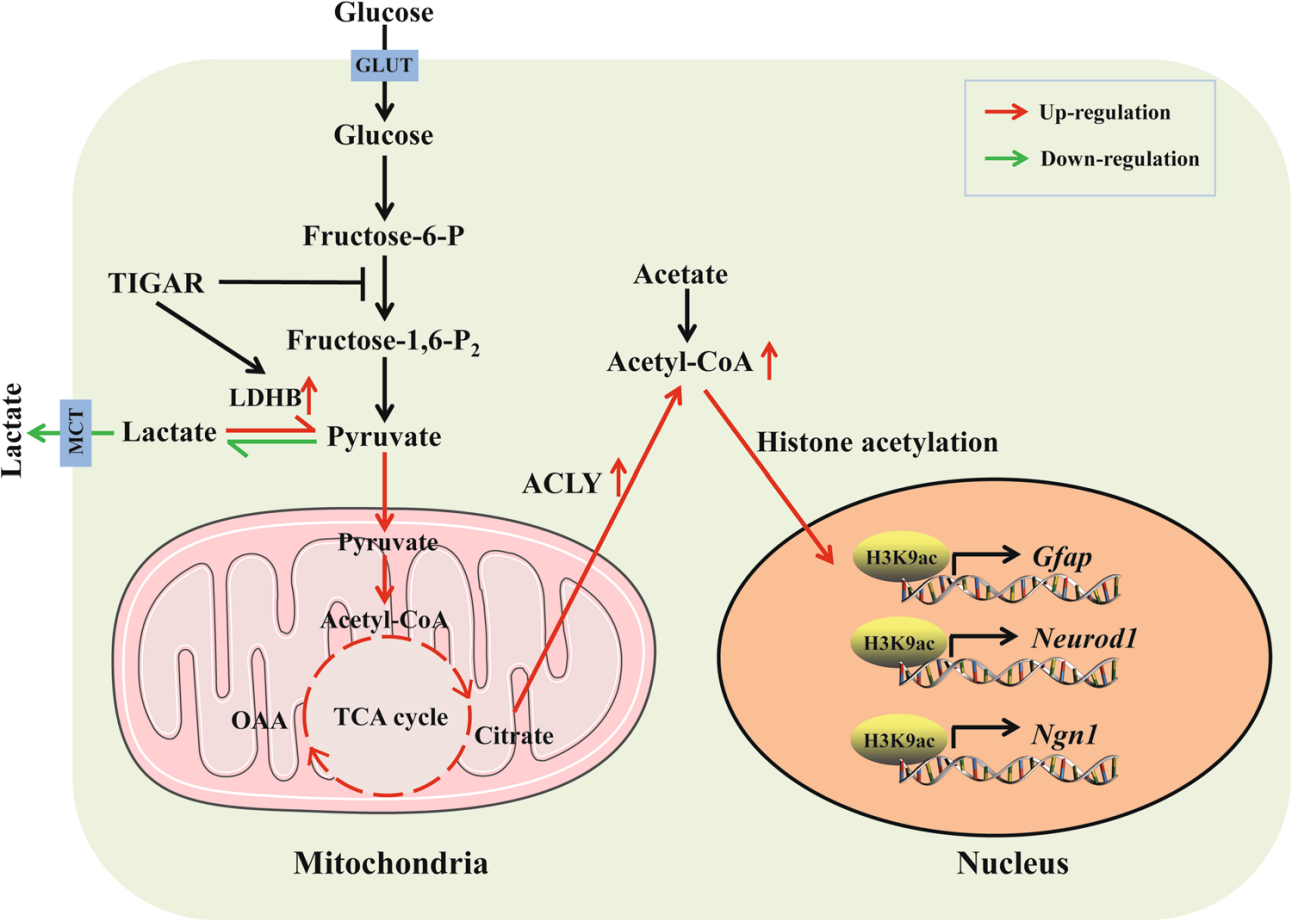
Schematic representation of TIGAR-induced neural differentiation of NSCs. TIGAR enhanced mitochondrial biogenesis and oxidative phosphorylation by increasing the expression of *Ldhb*, *Pgc-1α*, *Nrf1*, and *Mitoneet*, which triggers increases in acetyl-CoA and H3K9 acetylation. The upregulation of H3K9 acetylation resulted in the transcriptional activation of *Ngn1*, *Neurod1*, and *Gfap* during NSC differentiation

Metabolic reprogramming from aerobic glycolysis to OXPHOS is a hallmark of neuronal differentiation^4,12^. However, it remains unknown what triggers metabolism transition and how glucose metabolism directs neuronal differentiation. TIGAR, which acts as endogenous inhibitor of glycolysis and is widely distributed in neurons, attracted our interest. The results of this study provide several new insights into the effect of TIGAR on NSC differentiation. First, TIGAR is highly expressed in NSCs and increases as neural differentiation proceeds during embryonic cortex development. In vitro experiments further demonstrated that TIGAR is necessary and sufficient for NSC differentiation. Second, TIGAR promoted a shift in glucose metabolism to a mitochondrial phenotype and increased the levels of acetyl-CoA and H3K9 acetylation during NSC differentiation. Third, TIGAR might direct NSC differentiation via the upregulation of acetyl-CoA and H3K9 acetylation Fig. 7.

TIGAR is a potential candidate for metabolic regulation and directs neural differentiation. Glycolysis and mitochondrial OXPHOS are two major pathways that provide energy and biosynthetic precursors to cells. The balance between glycolysis and OXPHOS is essential for biological activity in cells. Regulation of metabolic programming is regarded as an important strategy to control cell fate and relieve injury caused by disease. In this study, TIGAR was selected as a potential target through which to regulate metabolism in NSCs. First, we found that the expression of TIGAR in the embryonic cortex was increased at E12.5–E16.5. Furthermore, immunostaining results demonstrated that TIGAR was highly expressed in NSCs in the VZ/SVZ and CP at E16.5. It has been reported that, during the development of the mouse neocortex, neurogenesis occurs around E12, peaks around E15 and finishes around birth^20^. Thus, TIGAR has the same expression pattern as the initiation of neural differentiation in the embryonic cortex.

Our in vitro experiments demonstrated that TIGAR is necessary and sufficient for NSC differentiation. Previous studies have reported that the levels of many metabolic enzymes changed dramatically during neural differentiation, including GLUTs, HK2, LDHA, and PKM^11,12,27^. However, we regard TIGAR as a regulator of glucose metabolism rather than merely a step in the process. TIGAR was initially identified as a p53-inducible gene. Studies have reported that p53 is a key factor in NSCs and can regulate neuronal differentiation at multiple levels^28,29^. In cancer cells, p53 inhibits glycolysis and restricts intracellular reactive oxygen species (ROS) levels through TIGAR and promotes OXPHOS by upregulating cytochrome c oxidase 2 (SCO2)^6,30^. According to our data, TIGAR may act as a very important bridge between p53 and metabolic reprogramming during NSC differentiation.

Furthermore, TIGAR accelerated glucose metabolism to a mitochondrial phenotype. Our data showed that TIGAR had no effect on the mRNA levels of *Gluts*, *Mcts*, *Pfkfb3*, or *Ldha* or on the uptake of glucose. However, knockdown of TIGAR decreased the expression of LDHB and enhanced lactate production during neural differentiation. LDHB converts lactate into pyruvate to be further oxidized in mitochondria^31^. This suggests that TIGAR may enhance OXPHOS by increasing the level of pyruvate via LDHB. Evidence has shown that overexpression of TIGAR in carcinoma cells increases the expression of PGC-1α, NRF1, MitoNEET, and Tomm20, which are important markers of mitochondrial biogenesis and OXPHOS^32^. Similarly, our data showed that TIGAR increased the expression of PGC-1α, NRF1, and MitoNEET, and enhanced oxygen consumption rate and ATP generation in differentiated NSCs. This suggested that TIGAR enforced a mitochondrial oxidative phosphorylation phenotype during NSC differentiation. In addition, acetyl-CoA, a key metabolite during OXPHOS, influences many cellular processes, including energy metabolism, autophagy, and cell proliferation and differentiation^23,33^. In this study, we found that knockdown of TIGAR reduced both whole cell and mitochondrial acetyl-CoA. Evidence has shown that acetyl-CoA can be generated from mitochondria-derived citrate by the cytosolic enzyme ACLY^23^. Our data showed that knockdown of TIGAR decreased the expression of ACLY. Citrate is an important intermediate in the tricarboxylic acid (TCA) cycle, and its abundance increases as mitochondrial OXPHOS is enhanced^34,35^. Citrate can be exported to the cytosol via the dicarboxylate antiporter solute carrier family 25, member 1 (SLC25A1), and converted into acetyl-CoA and oxaloacetate by ACLY^36^. In this study, blocking the metabolic enzyme activity of TIGAR abolished its effect on glycolysis and OXPHOS, and the expression of *Acly*. Therefore, we hypothesized that inhibition of mitochondrial OXPHOS and mitochondrial acetyl-CoA generation by knockdown of TIGAR might influence acetyl-CoA derived citric acid and the expression of ACLY.

Finally, TIGAR regulated neural differentiation through an epigenetic mechanism. Mounting evidences have shown that the production of acetyl-CoA is closely linked to the regulation of histone acetylation^23,37^. However, the effect of acetyl-CoA in NSCs is not clear. We demonstrated that TIGAR specifically regulated H3K9 acetylation during NSC differentiation. Furthermore, ChIP experiments showed that TIGAR enhanced the levels of H3K9 acetylation at the *Ngn1*, *Neurod1*, and *Gfap* promoters. These results demonstrated that TIGAR regulated the expression of genes related to NSC differentiation by increasing H3K9 acetylation. Acetate, a precursor of acetyl-CoA, rescued the decreases in H3K9 acetylation and MAP2 and GFAP expression resulting from knockdown of TIGAR. Thus, we demonstrated that TIGAR epigenetically directed NSC differentiation.

In summary, our work reveals a novel mechanism of TIGAR-regulated NSC differentiation. Future developmental clinical experiments may identify TIGAR as a potential target for the treatment of neurological diseases.

## Supplementary information

TIGAR and NSC differentiation supplementary material
