# Supplementary material for: TIGAR promotes neural stem cell differentiation through acetyl-CoA-mediated histone acetylation
Source: Cell Death Dis. 2019 Feb 27;10(3):198. doi: 10.1038/s41419-019-1434-3 (PMC6393469; doi:10.1038/s41419-019-1434-3)
Supplement: Supplementary file 1 — TIGAR and NSC differentiation supplementary material [file 41419_2019_1434_MOESM1_ESM.doc]

**Supplementary Materials**

**Title: TIGAR promotes neural stem cell differentiation through acetyl-CoA-mediated histone acetylation**

**Running title: TIGAR regulates neural stem cell differentiation**

**Authors:** Wenjuan Zhou^1^, Tiantian Zhao^1^, Jingyi Du^1^, Guangyu Ji^1^, Xinyue Li^1^, Shufang Ji^1^, Wenyu Tian^1^, Xu Wang^1^, Aijun Hao^1^,**^†^**

1. Key Laboratory of the Ministry of Education for Experimental Teratology, Shandong Provincial Key Laboratory of Mental Disorders, Department of Human Anatomy and Histoembryology, School of Basic Medical Sciences, Shandong University, Jinan, Shandong, China

**†**Corresponding author

**Contact:** [aijunhao@sdu.edu.cn](mailto:aijunhao@sdu.edu.cn)

**Contain:** Supplementary Figures S1-S2

**
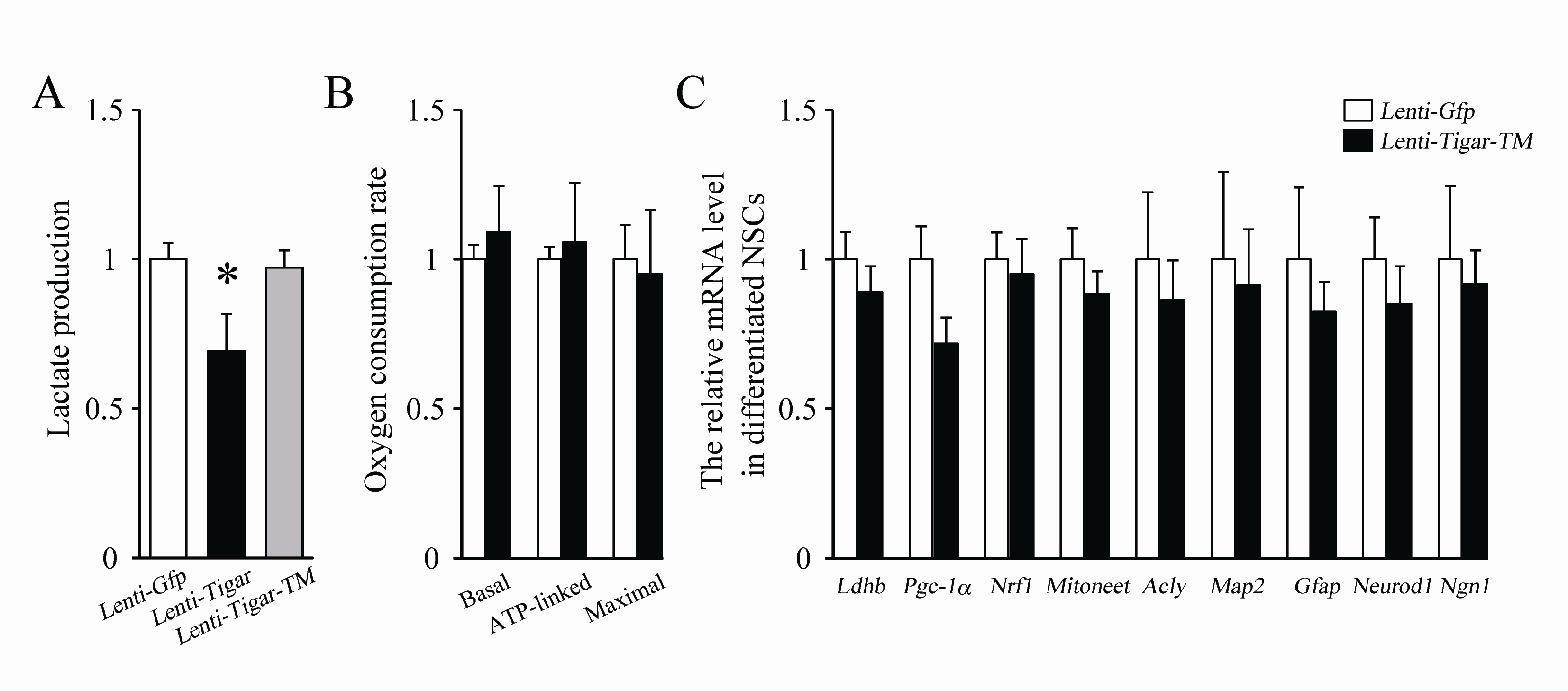
**

**Figure S1. Overexpression of TIGAR-TM showed no effect on oxidative phosphorylation and NSC differentiation.** According to previous study, we generated the *Tigar*-TM mutant (triple mutant H11A/E102A/H198A) to abolish TIGAR enzyme activity in FBPase-2. (A) Overexpression of *Tigar*-TM lost its inhibition of lactate production in differentiated NSCs. (B) Oxygen consumption rate (OCR) in Lenti-*Gfp*- and Lenti-*Tigar*-TM-treated NSCs. (C) The expression levels of *Ldhb*, *Pgc-1α*, *Nrf1*, *Mitoneet*, *Acly*, *Map2*, *Gfap*, *Neurod1* and *Ngn1* are not changed after treatment with *Lenti-Tigar-TM* in NSCs.

**
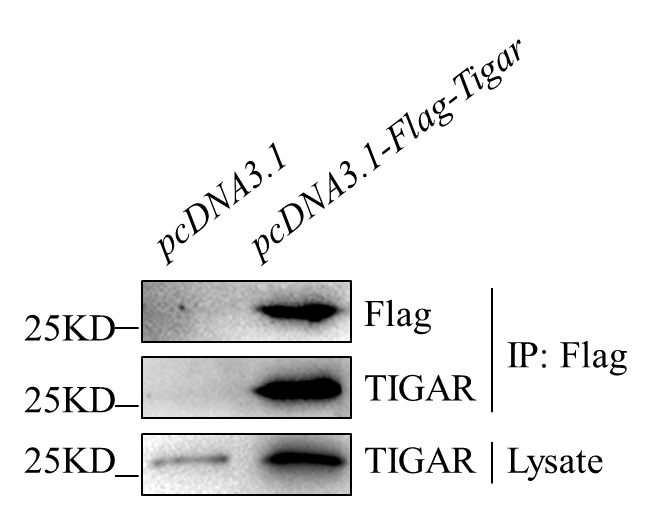
**

**Figure S2. TIGAR antibody (Abcam 37910) displayed specific reactivity to mouse TIGAR.** Briefly, we used immunoprecipitation (IP) assay to examine the activity to mouse TIGAR. HEK293 cells were transfected with pcDNA3.1-Flag-*mTigar* plasmid. The lysates were centrifuged and the supernatant was incubated with anti-Flag M2-conjugated beads. Finally, TIGAR antibody (Abcam 37910) was used for immunoblot analysis.
